# Serine Arginine-Rich Splicing Factor 1 (SRSF1) Contributes to the Transcriptional Activation of *CD3ζ* in Human T Cells

**DOI:** 10.1371/journal.pone.0131073

**Published:** 2015-07-02

**Authors:** Vaishali R. Moulton, Andrew R. Gillooly, Marcel A. Perl, Anastasia Markopoulou, George C. Tsokos

**Affiliations:** Division of Rheumatology, Department of Medicine, Beth Israel Deaconess Medical Center, Harvard Medical School, Boston MA, United States of America; University of Patras Medical School, GREECE

## Abstract

T lymphocytes from many patients with systemic lupus erythematosus (SLE) express decreased levels of the T cell receptor (TCR)-associated CD3 zeta (ζ) signaling chain, a feature directly linked to their abnormal phenotype and function. Reduced mRNA expression partly due to defective alternative splicing, contributes to the reduced expression of CD3ζ chain. We previously identified by oligonucleotide pulldown and mass spectrometry approaches, the serine arginine-rich splicing factor 1 (SRSF1) binding to the 3’ untranslated region (UTR) of *CD3ζ* mRNA. We showed that SRSF1 regulates alternative splicing of the 3’UTR of *CD3ζ* to promote expression of the normal full length 3`UTR over an unstable splice variant in human T cells. In this study we show that SRSF1 regulates transcriptional activation of *CD3ζ*. Specifically, overexpression and silencing of SRSF1 respectively increases and decreases *CD3ζ* total mRNA and protein expression in Jurkat and primary T cells. Using promoter-luciferase assays, we show that SRSF1 enhances transcriptional activity of the *CD3ζ* promoter in a dose dependent manner. Chromatin immunoprecipitation assays show that SRSF1 is recruited to the *CD3ζ* promoter. These results indicate that SRSF1 contributes to transcriptional activation of *CD3ζ*. Thus our study identifies a novel mechanism whereby SRSF1 regulates CD3ζ expression in human T cells and may contribute to the T cell defect in SLE.

## Introduction

Systemic lupus erythematosus (SLE) is a chronic debilitating disease, which afflicts mainly women in the childbearing years. Dysfunctional cellular and antibody responses contribute to the etio-pathogenesis of this autoimmune disease. Autoantibodies and immune complexes deposit in target tissues such as kidneys and lead to organ failure. T lymphocytes play an important role in SLE pathophysiology–not only do they provide cognate help to autoreactive B cells, but also contribute to cytokine imbalance and infiltrate target organs leading to tissue damage [1, 2].

T cells from a number of SLE patients express numerous defects in signaling including reduced amounts of the T cell receptor (TCR) associated CD3 zeta (ζ) signaling chain [3–6]. A rewiring of the TCR in SLE T cells involves replacement of CD3ζ chain by the homologous Fc epsilon receptor I gamma (FcRγ) signaling chain [7] which recruits the spleen tyrosine kinase (Syk) instead of the zeta associated protein of 70kD (ZAP-70) normally recruited by the ζ chain [8]. The FcRγ-Syk interaction is manifold stronger than the Zeta—ZAP-70 bond thus leading to a stronger downstream intracellular signal as evidenced by the increased tyrosine phosphorylation of signaling intermediates and increased calcium flux which contributes to the hyperexcitable phenotype of the SLE T cell [9]. Despite this hyperactive signaling, lupus T cells fail to produce sufficient amounts of the crucial cytokine IL-2. We showed that forced expression of the CD3ζ chain into SLE T cells led to correction of the abnormal signaling i.e., tyrosine phosphorylation and calcium fluxing and more importantly restoration of IL-2 production [10]. These studies indicate a critical role of the CD3ζ chain in the abnormal T cell phenotype in SLE patients. However the mechanisms leading to the decreased expression of CD3ζ chain in SLE are not fully understood.

We previously described a defective alternative splice variant of the *CD3ζ* 3`UTR expressed in SLE T cells, which renders the *CD3ζ* mRNA unstable thus leading to reduced expression of CD3ζ chain protein [11]. We recently identified by mass spectrometry of Jurkat cell nuclear proteins, the serine arginine-rich splicing factor 1 (SRSF1) or splicing factor 2 / alternative splicing factor (SF2/ASF) pulled down by a *CD3ζ* mRNA oligonucleotide. We showed that SRSF1 regulates alternative splicing of the *CD3ζ* 3`UTR such that it promotes expression of the full length isoform over the defective splice variant, thus promoting the normal expression of CD3ζ chain in human T cells [12, 13]. We showed that T cells from SLE patients express reduced levels of SRSF1, more so patients with worse disease, as determined by their SLE disease activity index (SLEDAI) [14].

SRSF1, a prototype member of the serine arginine (SR) family of splicing proteins is a well-recognized regulator of alternative splicing. A predominantly nuclear protein, SRSF1 can shuttle between the nucleus and cytoplasm [15]. While SR proteins are mainly known for their role in regulating gene expression at the post-transcriptional level–such as mRNA splicing [16], stability [17], and translation [18], they have been recently recognized to play a role in transcriptional regulation, via interactions with the basal transcription machinery [19]. SR proteins SRSF1 and SRSF2 were shown to accumulate at gene promoters via interactions within the 7SK small nuclear ribonucleoprotein (7SK snRNP) complex, with SRSF2 shown to play a direct role in transcriptional activation, and SRSF1 and others to indirectly activate transcription [20, 21]. A handful of endogenous target genes such as Caspase 9, Bcl-x [22], and CD45 [23] are described to be regulated by SRSF1, however its precise role in T cell physiology, is not known. Interestingly, we recently demonstrated that forced expression of SRSF1 into T cells from SLE patients rescued IL-2 production, and mediated an increase in *IL-2* mRNA expression and transcriptional activation [14].

In this study, we asked whether SRSF1 contributes to the regulation of *CD3ζ* gene expression at the level of transcription. We show here that SRSF1 increases the expression of *CD3ζ* mRNA in primary human T cells. Forced expression of SRSF1 in T cells leads to increased transcriptional activity of the *CD3ζ* promoter in a dose dependent manner, and SRSF1 is recruited to the *CD3ζ* promoter. Furthermore, the expression of SRSF1 correlates with the CD3ζ chain expression in T cells from patients with SLE.

## Materials and Methods

### Human subjects

Patients fulfilling the criteria of the American College of Rheumatology (ACR) for the classification of SLE [24], and control healthy individuals (age, race and gender matched) were recruited at the Beth Israel Deaconess Medical Center (BIDMC) Rheumatology clinic. Peripheral blood samples were obtained by venipuncture. Written informed consent was obtained from all subjects. Peripheral blood samples from healthy adult volunteers were also obtained from the Kraft donor center of the Dana Farber Cancer Institute, and the blood donor center at Boston Childrens hospital, Boston, MA.

### Ethics statement

Written informed consent was obtained from all subjects. All study protocols were approved by the Beth Israel Deaconess Medical Center (BIDMC) institutional review board (IRB).

### Cells, plasmids and reagents

T cells were purified from peripheral blood using the Rosette Sep T cell purification kit (Stem Cell Technologies Inc., Vancouver, CA). Jurkat cells (clone E6-1) and 293T cells were purchased from American Type Culture Collection (ATCC, Manassas, VA). The pcDNA3.1-SRSF1-HA plasmid was a gift from Dr. James Manley (Columbia University, NY). The pGL2-zeta promoter-luciferase constructs were a gift from Dr. Barbara Rellahan [25, 26] and pGL2-basic vector from Promega (Madison, WI). SRSF1 antibody was purchased from Life technologies (Carlsbad, CA), CD3ζ χλoνε 6B.10) and horseradish peroxidase (HRP)-conjugated antibodies were from Santa Cruz Biotechnology (Santa Cruz, CA). β-actin antibody was purchased from Sigma-Aldrich (St. Louis, MO). SRSF1 specific siRNA and control siRNA were purchased from Qiagen (Valencia, CA)

### Transfections

Transient transfections in primary human T cells and Jurkat cells were performed using the Lonza nucleofector system (Lonza, Cologne, Germany). Briefly, 5 x 10^6^ cells were resuspended in 100μL of the human T cell nucleofector solution, then plasmid DNA (0.5μg/10^6^ cells) or siRNA was added and cells were transfected in a cuvette using the nucleofector program U-014 for primary cells and X-001 for Jurkat cells. Cells were rescued immediately in prewarmed complete RPMI medium (supplemented with 10% fetal bovine serum and 1% penicillin and streptomycin antibiotics) in 24 well plates and incubated in a 37°C CO2 incubator. Transfections in 293T cells were performed using Lipofectamine 2000 (Life technologies) according to manufacturer’s instructions.

### Western blotting

Cells were pelleted and lysed with radioimmunoprecipitation assay (RIPA) buffer (Boston Bioproducts, MA). Total protein lysates were resolved on 4–12% Bis-tris gels, and transferred to polyvinylidene difluoride (PVDF) membrane. Membranes were blocked with 5% non-fat milk in Tris buffered saline with 0.05% Tween 20 (TBS-T) for 1 hour, incubated with primary antibody (1:1000) overnight at 4°C, washed thrice with TBS-T, incubated with HRP conjugated secondary antibody (1:2000) for 1 hour, washed thrice with TBS-T, developed with enhanced chemiluminescence (ECL) reagents (GE Healthcare, Piscataway, NJ) and visualized with a Fujifilm LAS-4000 imager. Densitometric quantitation of western blot images was performed using the Bio-Rad Quantity One software (Bio-Rad Labs, Hercules, CA).

### mRNA expression studies

Total RNA was isolated using the RNeasy mini kit (Qiagen, Valencia, CA). 200ng of total RNA was reverse transcribed into single stranded cDNA using the oligo-dT primed Ecodry RNA to cDNA premix (Clontech, Mountain View, CA). Real time quantitative PCR amplification was carried using the Sybr green I mastermix in a LightCycler 480 (Roche, Germany) as follows: initial denaturation at 95°C for 5 minutes, 40 cycles of amplification: denaturation at 95°C for 15 seconds, annealing at 60°C for 15 seconds, and extension at 72°C for 30 seconds; 1 cycle of melting curves at 95°C for 15 seconds, 65°C for 2 minutes and 97°C continuous and a final cooling step at 37°C for 30 seconds. Threshold cycles (Ct) values were used to calculate relative mRNA expression by the ΔCt relative quantification method. Primer sequences are as follows—*CD3ζ* forward 5`- TGC TGG ATG GAA TCC TCT TC -3`and reverse 5`- CCG CCA TCT TAT CTT TCT GC -3`and housekeeping gene *Cyclophilin A*: forward 5′- TTC ATC TGC ACT GCC AAG AC-3′ and reverse 5′- TCG AGT TGT CCA CAG TCA GC -3′.

### Luciferase assays

Five hundred thousand 293T cells were co-transfected with 0.5μg of the pGL2-*CD3 zeta* promoter—luciferase reporter construct and increasing amounts (1μg, 1.5μg, 2.5μg) of effector plasmid pcDNA3.1 or pcDNA3.1-SRSF1 for effector: reporter plasmid ratios of 2:1, 3:1 and 5:1. Each transfection included 25ng of the pRL-TK *Renilla* luciferase construct as an internal control. Twenty-four hours after transfection, luciferase activity was quantified using the dual-luciferase assay system (Promega, Madison, WI) according to manufacturer’s instructions. Five million primary human T cells were co-transfected with 0.8μg of the pGL2-*CD3 zeta* promoter—luciferase reporter construct and increasing amounts (2.4μg, 3.2μg, 4μg) of effector plasmid (either pcDNA3.1 or pcDNA3.1-SRSF1) to obtain effector: reporter plasmid ratios of 3:1, 4:1, and 5:1. Each transfection included 25ng of the pRL-TK *Renilla* luciferase construct as an internal control. Four hours after transfection, cells were collected, lysed and luciferase activity was quantified using the dual-luciferase assay system

### Reporter Chromatin Immunoprecipitation (R-ChIP) assays

293T or primary T cells were co-transfected with the human *CD3ζ* promoter luciferase construct (0.5 μg/10^6^ cells) and either empty vector or SRSF1 expression vector (1.5 μg /10^6^ cells). 24h (293T cells) or 4h (primary T cells) post transfection, cells were collected and the assay performed using the MAGnify ChIP kit (Life technologies, Grand Island, NY) according to the manufacturer’s instructions. Briefly, cells were fixed for 10 min in 1% formaldehyde to cross-link DNA-protein and protein-protein complexes, and Glycine (1.25M) added for 5min to stop cross-linking. Cells were washed with cold phosphate-buffered saline, resuspended in lysis buffer containing protease inhibitors and sonicated to shear DNA, pelleted, and diluted supernatants were immunoprecipitated with the indicated antibodies overnight. 10% of the diluted supernatants were kept as “input” for normalization. After several washing steps, bound protein was digested with proteinase K, and the cross-linking reversed at 65°C. DNA was purified and amplified by quantitative PCR on a Lightcycler 480 (Roche, Germany), using specific primers flanking the *CD3 zeta-3* promoter at position -65 (forward primer 5`- CCA CAG TCC TCC ACT TCC TG - 3`) and the luciferase gene (reverse primer 5`- GGA GAG CAA CTG CAT AAG GC - 3`). Threshold cycle (Ct) values were used to calculate the proportion of immunoprecipitated chromatin against input chromatin by the ΔCt relative quantification method. ChIP signals from specific antibodies (anti-HA or anti-SRSF1) were normalized to those of control IgG antibody.

### Statistical analysis

Statistical analyses were performed using the Student’s *t*-test, Mann-Whitney test (for non parametric analysis), and the Spearman R correlation coefficient using the GraphPad Prism software. A *p* value <0.05 was considered statistically significant.

## Results

### SRSF1 expression correlates with CD3ζ expression in T cells from patients with SLE

**Fig 1:**
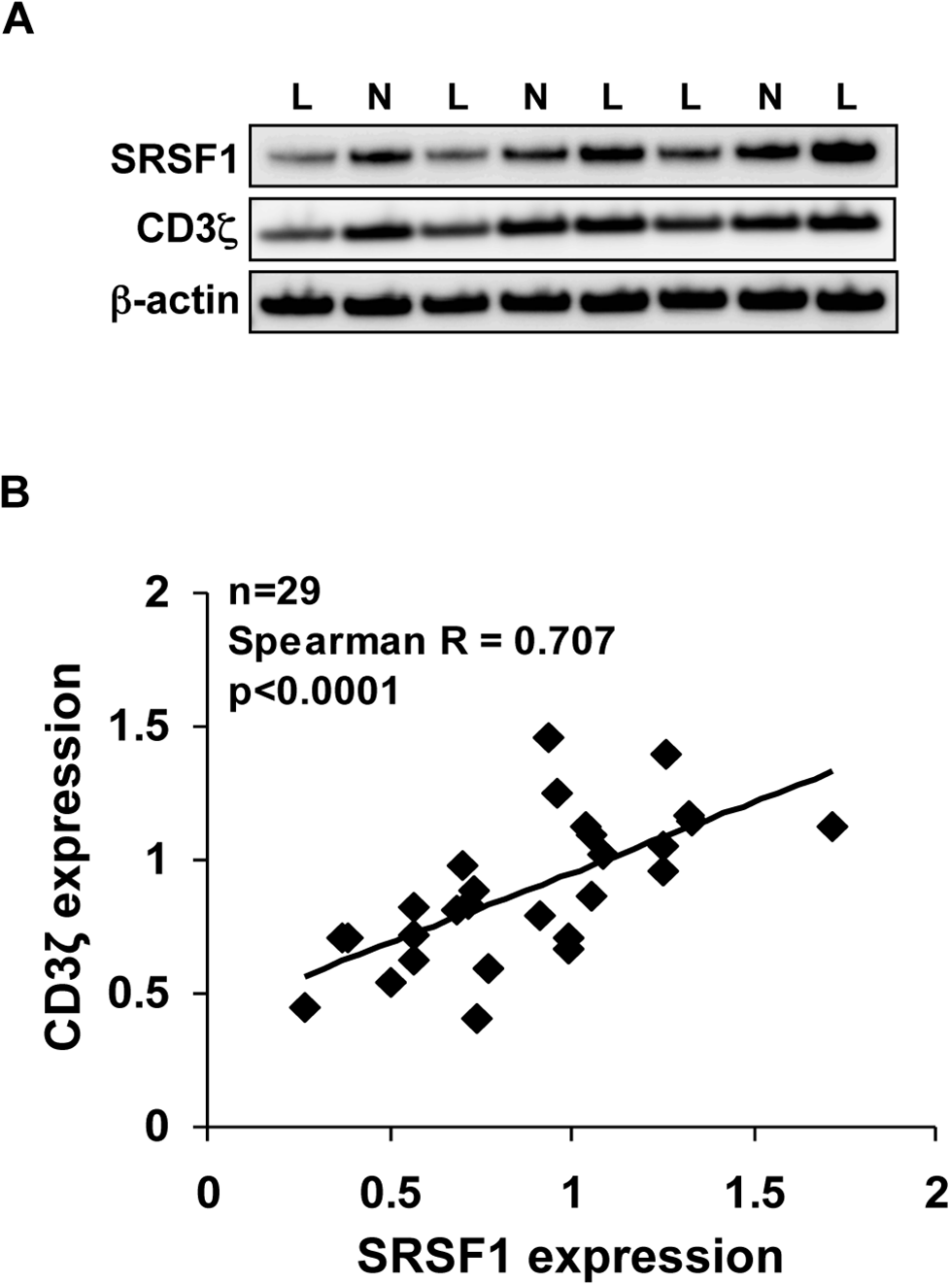
Correlation between SRSF1 and CD3ζ chain expression in T cells from patients with SLE. (A) Peripheral blood T cells from SLE patients (“L”, n = 29) and healthy individuals (“N”, n = 23) were lysed and total protein immunoblotted for SRSF1, CD3ζ and β-actin. Representative blots are shown. (B) Densitometric quantitation of SRSF1 and CD3ζ blots were performed and normalized to β-actin. SRSF1 and CD3ζ expression of SLE patients were normalized to those from healthy individuals and the relative values were plotted on an x/y graph.

T cells from patients with SLE express altered amounts of the T cell receptor associated CD3ζ signaling chain, such that several patients exhibit reduced expression of this signaling chain. We previously performed mass spectrometry analysis of nuclear proteins pulled down by an RNA oligonucleotide corresponding to the 3`untranslated region (UTR) of *CD3ζ*. We identified the serine arginine-rich splicing factor 1 (SRSF1) in that analysis and confirmed SRSF1 binding to the 3`UTR of *CD3ζ* mRNA. We showed in T cells isolated from a small number of SLE patients a direct correlation between SRSF1 and CD3ζ protein expression [13]. Here, in a substantially increased sample size (29 SLE patients and 23 healthy individuals), we examined SRSF1 and CD3ζ chain protein expression in T cells from peripheral blood of SLE patients (“L”) and age-, race- and gender-matched healthy (“N”) individuals (Fig 1A). Immunoblots for SRSF1 and CD3ζ were quantified by densitometry, and normalized to β-actin loading control. Expression from SLE patients was normalized to the expression level in respective matched healthy individuals and relative values depicted on an xy plot (Fig 1B). Data show that the SRSF1 protein expression significantly correlates with CD3ζ chain expression in SLE T cells with a correlation coefficient Spearman R = 0.707, and p value <0.0001 (Fig 1B).

### SRSF1 regulates total mRNA expression of CD3ζ

**Fig 2:**
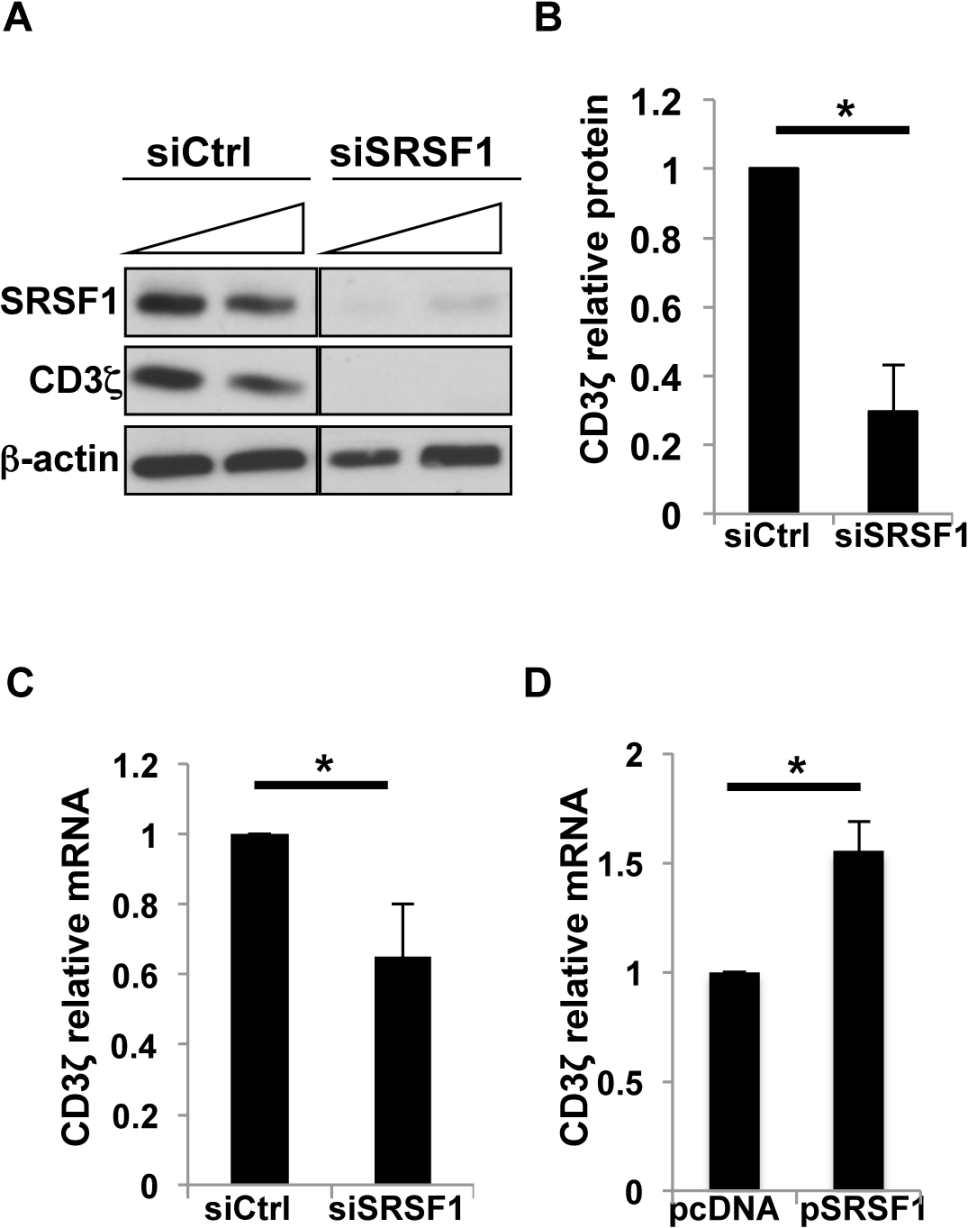
SRSF1 regulates CD3ζ protein and mRNA expression. (A) Jurkat cells were transfected with increasing concentrations of SRSF1 specific siRNA (siSRSF1) or non-silencing control siRNA (siCtrl). 24–48 hrs post transfection, cells were lysed and total protein immunoblotted for SRSF1, CD3ζ and β-actin. (B) Densitometric quantitation of SRSF1 and CD3ζ blots was performed and normalized to β-actin. Graphs show average values from three independent experiments and error bars represent SEM. (C) Primary T cells were transfected with siSRSF1 or siCtrl siRNA, and cells were collected 24hrs post transfection. Total RNA was reverse transcribed and quantitative real time pcr performed using specific primers for *CD3ζ* and *Cyclophilin A*. Graphs show average values from five independent experiments and error bars represent SEM. (D) Primary T cells were transfected with SRSF1 expression plasmid (pSRSF1) or an empty plasmid as control (pcDNA). Cells were collected 24hrs post transfection. Total RNA was reverse transcribed and quantitative real time pcr performed using specific primers for *CD3ζ* and *Cyclophilin A*. Graphs show average values from nine independent experiments and error bars represent SEM. Asterisks indicate p <0.05.

We previously showed that SRSF1 regulates alternative splicing of the *CD3ζ* 3`UTR mRNA such that it promotes expression of the full length 3`UTR over a truncated splice variant [13]. SRSF1 and related SR family member SRSF2 have recently been suggested to activate gene transcription via interactions with the basal transcription machinery [19]. Based on these concepts, we asked whether SRSF1 also regulates *CD3ζ* transcription. We first confirmed that the silencing of SRSF1 in the Jurkat human T cell line led to the downregulation of CD3ζ protein expression. We used siRNA specific for SRSF1 (siSRSF1) or control non-silencing siRNA (siCtrl), and found that SRSF1 knockdown led to corresponding decrease in CD3ζ chain expression (Fig 2A and 2B). In Fig 2B, data show average values from three independent experiments (1 ± 0 vs 0.29 ± 0.25, p = 0.01). To ask whether SRSF1 regulates total *CD3ζ* mRNA expression in an endogenous system, we silenced SRSF1 expression in primary T cells, and using primers specific for the coding region of *CD3ζ*, assessed the total expression of *CD3ζ* mRNA. We observed a significant decrease in *CD3ζ* mRNA levels in siSRSF1-transfected cells compared to controls (Fig 2C). In Fig 2C, data show average values from five independent experiments (1 ± 0 vs 0.65 ± 0.2, p = 0.04). In parallel, we overexpressed SRSF1 using an expression vector (pSRSF1) or empty plasmid (pcDNA) as control, and observed that SRSF1 overexpression led to increased *CD3ζ* mRNA levels in human T cells (Fig 2D). In Fig 2D, data show average values from nine independent experiments (1 ± 0 vs 1.55 ± 0.05, p = 0.001). These results show that SRSF1 enhances total *CD3ζ* mRNA expression levels.

### SRSF1 increases activity of the *CD3ζ* promoter

**Fig 3:**
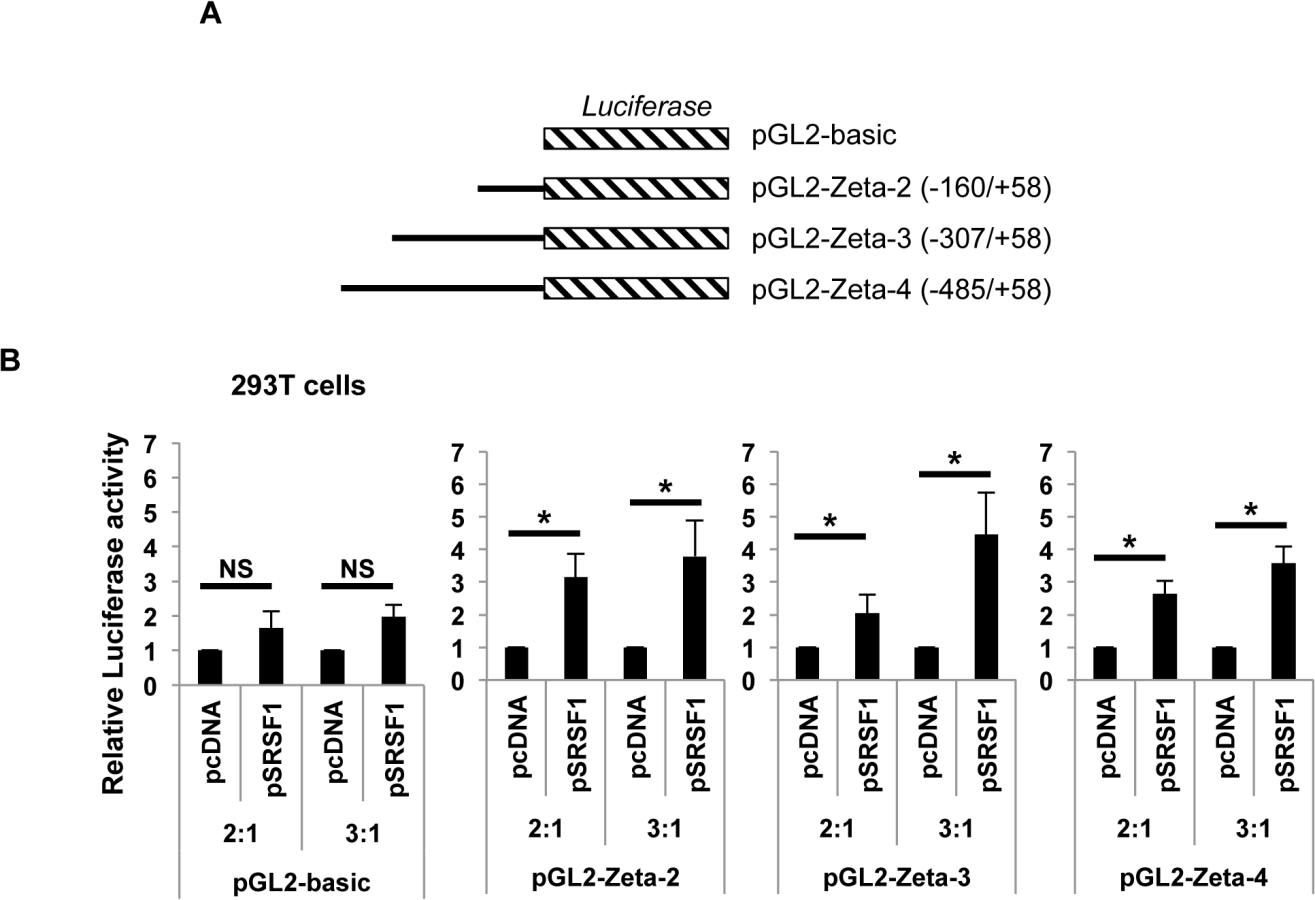
SRSF1 regulates transcriptional activity of the *CD3ζ* promoter in 293T cells. (A) Schematic showing luciferase constructs with different lengths of the *CD3ζ* promoter. (B) 293T cells were co-transfected with an empty pGL2-basic plasmid or the indicated CD3ζ promoter-luciferase constructs and empty vector (pcDNA) or SRSF1 expression vector (pSRSF1) in increasing concentrations such that the ratio of the pcDNA or pSRSF1 (i.e., effector) plasmid to the luciferase (i.e., reporter) plasmid was 2:1 or 3:1. 24hours post transfection, cells were lysed, and luciferase activity measured using the dual-luciferase assay. Graphs show average values from at least three independent experiments and error bars represent SEM. Asterisks indicate p <0.05; NS = not significant.

**Fig 4:**
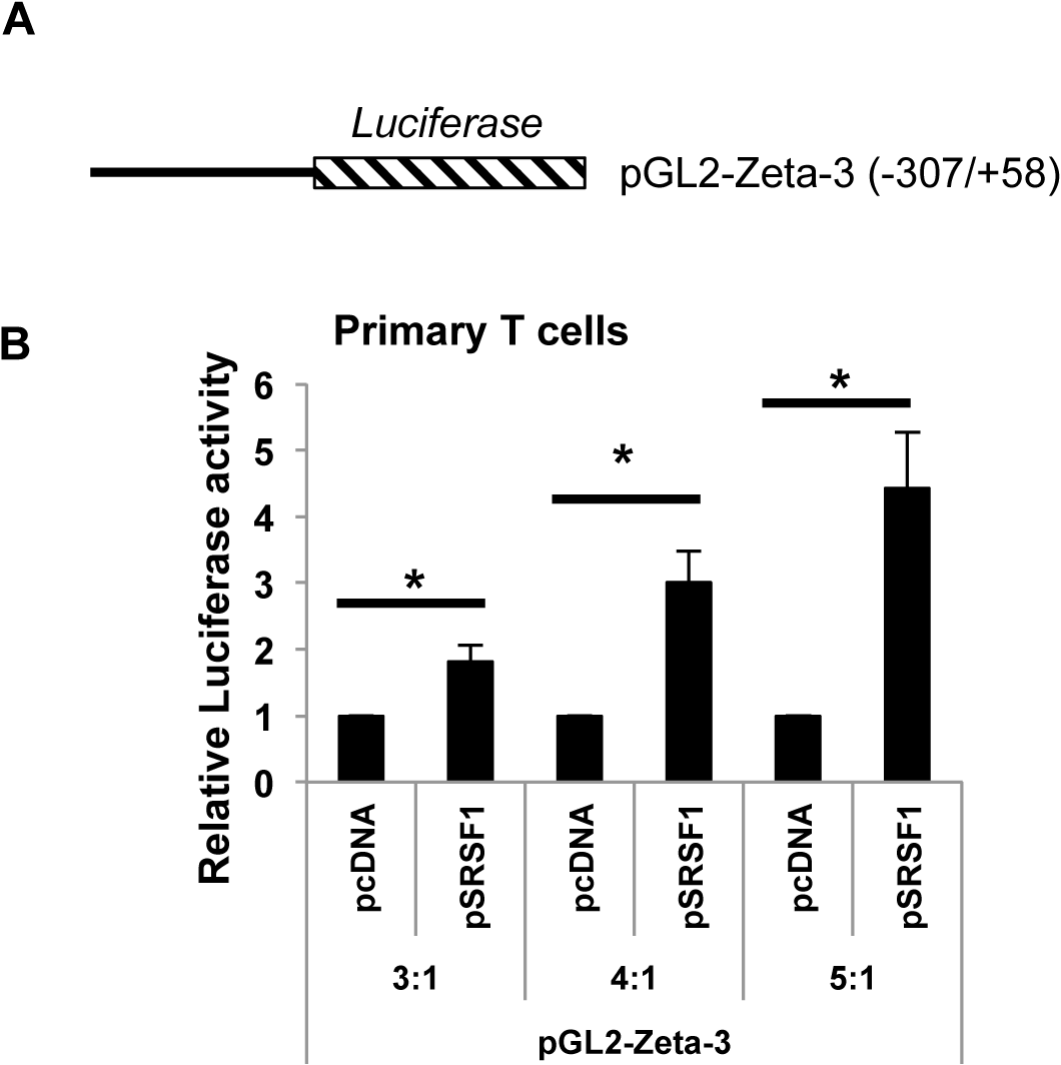
SRSF1 regulates transcriptional activity of the *CD3ζ* promoter in primary human T cells. (A) Schematic showing the *CD3ζ* promoter-luciferase (pGL2-Zeta-3) construct. (B) Peripheral blood T cells were transfected with the pGL2-Zeta-3 reporter construct and co-transfected with either empty vector (pcDNA) or an SRSF1 expression vector (pSRSF1) in increasing concentrations such that the ratio of the pcDNA or pSRSF1 (i.e., effector) plasmid to the luciferase (i.e., reporter) plasmid was 3:1, 4:1, or 5:1. 4 hours post transfection, cells were lysed and luciferase activity measured using the dual-luciferase assay. Graphs show average values from three independent experiments and error bars represent SEM. Asterisks indicate p <0.05.

In order to examine if SRSF1 can control transcriptional activity of *CD3ζ*, we performed promoter-luciferase reporter assays. We used three promoter-luciferase constructs (zeta-2, zeta-3 and zeta-4) with varying lengths (-160/+58, -307/+58 and -485/+58) of the *CD3ζ* promoter as shown in schematic in Fig 3A, according to the numbering published previously [26]. 293T cells were transfected with the *CD3ζ* promoter-luciferase ‘reporter’ construct and co-transfected with increasing amounts of the SRSF1 or empty ‘effector’ constructs such that effector: reporter plasmid ratios were 2:1, or 3:1, and promoter activity measured using dual-luciferase assays. As seen in Fig 3B, SRSF1 induced a dose dependent increase in *CD3ζ* promoter activity with all three lengths of the *CD3ζ* promoter (Fig 3B). As control, an empty luciferase construct (pGL2-basic) was co-transfected with increasing amounts of SRSF1. Data show average values from at least three independent experiments, error bars represent SEM Data for control vs SRSF1-transfected cells for the pGL2-basic construct were 1 ± 0 vs 1.65 ± 0.47 and 1.97 ± 0.35, p = 0.2 and 0.05, for the pGL2-Zeta-2 construct 1 ± 0 vs 2.47 ± 0.73 and 3.79 ± 1.1, p = 0.01 and 0.04, for the pGL2-Zeta-3 construct 1 ± 0 vs 2.05 ± 0.55 and 4.45 ± 1.27, p = 0.01 and 0.03, and for the pGL2-Zeta-4 construct 1 ± 0 vs 2.64 ± 0.39 and 3.57 ± 0.5, p = 0.02 and 0.01 for the 2:1 and 3:1 conditions respectively. To confirm these findings in primary T cells, we performed promoter-reporter assays in peripheral blood T cells. We co-transfected the -307/+58 construct (Fig 4A) with increasing amounts of SRSF1 (pSRSF1) or empty plasmid (pcDNA) as control (Fig 4B). We found significant dose dependent increase in *CD3ζ* promoter activity in the SRSF1 transfected cells compared with control-transfected cells (Fig 4B, 1 ± 0 vs 1.82 ± 0.23, 3 ± 0.48, 4.43 ± 0.83 and p = 0.03, 0.02 and 0.02 respectively for the 3:1, 4:1 and 5:1 conditions). Data show average values from three independent experiments, error bars represent SEM. These results indicate that SRSF1 positively regulates transcriptional activity of the *CD3ζ* promoter.

### SRSF1 is recruited to the *CD3ζ* promoter

**Fig 5:**
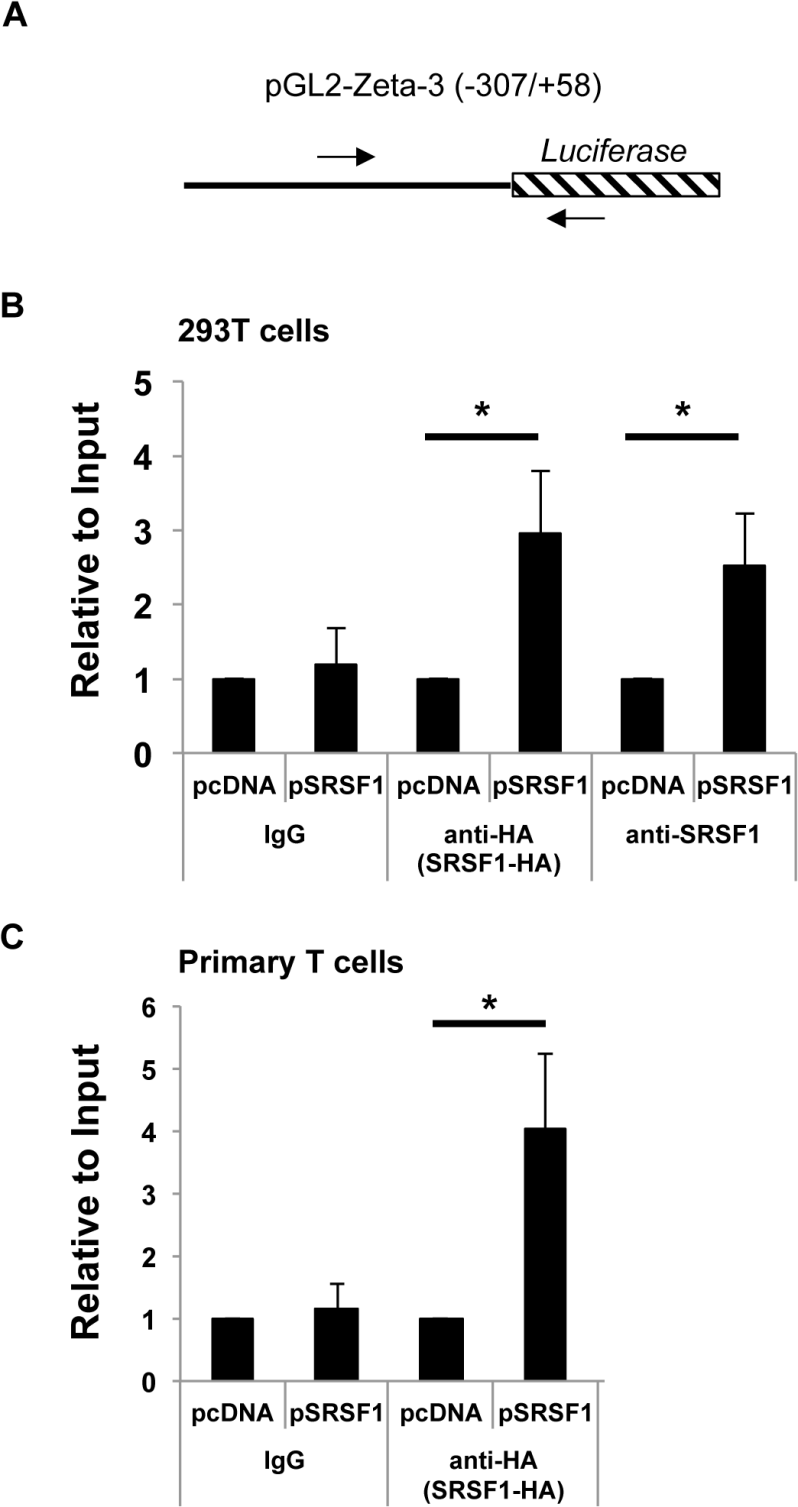
SRSF1 is recruited to the *CD3ζ* promoter. (A) Schematic showing the *CD3ζ* promoter-luciferase pGL2-Zeta-3 construct. Arrows indicate primers used for ChIP pcr amplification. (B) 293T cells were co-transfected with the *CD3ζ* promoter-luciferase construct and empty vector (pcDNA) or SRSF1 expression vector (pSRSF1). At 24hrs post transfection, cells were collected and ChIP assays performed using an SRSF1- specific antibody, HA-tag specific antibody or a control IgG antibody. The CD3ζ promoter-luciferase region was amplified by quantitative real time pcr and normalized to the input DNA. Graphs show average values from at least three independent experiments and error bars represent SEM. (C) Primary T cells were co-transfected and ChIP assays performed as in A, using an HA-tag specific antibody or a control IgG antibody. The CD3ζ promoter-luciferase region was amplified by quantitative real time pcr and normalized to the input DNA. Graphs show average values from at least four independent experiments and error bars represent SEM. Asterisks indicate p <0.05.

Based on our findings that SRSF1 increases the transcriptional activity of *CD3ζ* (Figs 3 and 4), and because it was previously shown that SR proteins through interactions with the basal transcription machinery could be recruited to the promoter region of target genes [19], we asked whether SRSF1 is recruited to the *CD3ζ* promoter. We performed reporter-chromatin immunoprecipitation (ChIP) assays after co-transfecting 293T cells with pcDNA or pSRSF1 expression plasmids and the -307/+58 *CD3ζ* promoter-luciferase reporter construct. The pSRSF1 cDNA expresses a hemagglutinin (HA) tag, therefore we used either an anti-SRSF1 antibody or an anti-HA tag antibody to immunoprecipitate SRSF1, and a control IgG antibody as negative control. Using primers spanning the *CD3ζ* promoter and the luciferase gene (Fig 5A) we amplified promoter DNA enriched in immunoprecipitates and normalized to those from input samples. As seen in Fig 5B we found an increased recruitment of SRSF1 to the *CD3ζ* promoter-luciferase construct in the SRSF1 transfected cells as compared to control transfected cells. Data show average values from at least three independent experiments (1 ± 0 vs 1.18 ± 0.48, 2.95 ± 0.84 and 2.52 ± 0.69 and p values 0.7, 0.04 and 0.02 for IgG, HA and SRSF1 respectively).

To confirm our ChIP findings in an endogenous system, we assessed SRSF1 recruitment to the *CD3ζ* promoter construct in primary T cells. We transfected peripheral blood T cells with the SRSF1 expression vector or an empty vector along with the *CD3ζ* promoter-luciferase construct (Fig 5C). Using an anti-HA tag antibody to immunoprecipitate SRSF1, and an IgG antibody as negative control, immunoprecipitates were analyzed for enrichment of the *CD3ζ* promoter. We found that SRSF1 was recruited to the *CD3ζ* promoter in cells transfected with SRSF1 compared to control cells, and control antibody immunoprecipitates (Fig 5C). Data show average values from at least four independent experiments (1 ± 0 vs 1.17 ± 0.37 and 4.03 ± 1.19 and p values 0.7 and 0.04 for IgG and HA respectively).

Altogether, these data suggest that SRSF1 is recruited to the *CD3ζ* promoter and contributes to transcriptional activation of *CD3ζ* in human T cells.

## Discussion

In this study we present several novel findings—we show that the splicing factor SRSF1 positively regulates total protein and mRNA expression of *CD3ζ*, second we show that SRSF1 regulates transcriptional activity of the CD3*ζ* promoter and finally that SRSF1 is recruited to the *CD3ζ* promoter. Additionally SRSF1 expression correlates with CD3*ζ* expression in SLE T cells. Taken together with our previous findings that SRSF1 regulates alternative splicing of *CD3ζ* 3`UTR, these findings suggest that SRSF1 regulates CD3ζ chain expression in human T cells through multiple mechanisms, and may contribute to the T cell molecular defect in SLE.

Although the SR protein family is classically thought to control gene regulation events at the post-transcriptional level, such as alternative splicing and mRNA transport, recently they have been identified to control steps in transcriptional regulation. It was shown that knocking out the SR splicing proteins SRSF1 and SRSF2 in the murine embryonic fibroblast (MEF) cell line led to a decrease in total nascent mRNA, suggesting their role in transcriptional activation [19]. SRSF2 was shown to be critical for recruitment of positive transcription elongation factor b (pTEFb) kinase, which is an essential step to release “paused” RNA polymerase II on the target gene, and thereby to regulate transcription elongation. More recently ChIP-seq analysis in MEF cells has shown that SRSF1 and SRSF2 are recruited to numerous gene promoters near the transcription start site through distinct interactions with promoter associated DNA and nascent RNA. Specifically SRSF1 and SRSF2 were shown to be part of the 7SK snRNP complex, a multi subunit ribonucleoprotein particle, and promoter associated nascent RNA [20]. Interactions of SR splicing factors with histones/chromatin have also been described. In a mass spectrometry analysis of protein-protein pull-down of nuclear extracts, linker histone H1 was shown to interact with numerous core splicing factors including SRSF1 [27]. Furthermore, depletion of SRSF1 led to heterochromatin protein 1 (HP1) proteins accumulation at mitotic chromatin and a delay in G0/G1 phase entry suggesting that SRSF1 may function as a chromatin sensor and is required for appropriate cell-cycle progression [28]. Our findings that SRSF1 is recruited to the *CD3ζ* promoter (Fig 5B and 5C) suggest that SRSF1 may be involved in *CD3ζ* transcriptional activation via similar mechanisms. Furthermore, because the *CD3ζ* promoter construct (-307/+58) bears two ets like factor (Elf)-1 binding sites, previously shown to be necessary for the transcriptional activation of the promoter [25, 26], it is possible that SRSF1 binds to the promoter indirectly through Elf-1. More work is needed to specifically elucidate which components of the transcriptional machinery associate with SRSF1 within human T cells.

We have previously shown that SRSF1 expression is altered in T cells from patients with SLE, such that patients with worse disease express reduced levels of this protein as compared to those with mild disease [14]. CD3ζ expression is controlled by multiple factors at the transcriptional [29, 30], post transcriptional [3, 12, 13] and post translational [31, 32] levels. At the protein level, increased ubiquitination and protein degradation of CD3ζ chain was observed in T cells from SLE patients [33]. Interestingly, SRSF1 undergoes ubiquitination and proteasome degradation during normal T cell activation, and exhibits increased ubiquitination in T cells from SLE patients compared with healthy individuals [34]. Our finding of a direct correlation between the protein expression of SRSF1 and CD3ζ in SLE T cells (Fig 1) not only reflects the SRSF1 mediated regulation of *CD3ζ*, but also common regulatory mechanisms that may be dysregulated in SLE.

In summary, we show here that SRSF1 contributes to *CD3ζ* transcriptional activation and thus CD3ζ chain protein expression, and decreased SRSF1 expression may account for the reduced CD3ζ chain expression in SLE T cells. Understanding the molecular mechanisms that contribute to T cell malfunction in patients with SLE should help design approaches to correct them in a targeted manner.
